# Prediction of blood-brain barrier-penetrating peptides using B3BPFN

**DOI:** 10.3389/fmolb.2026.1858506

**Published:** 2026-05-20

**Authors:** Xingchen Liu, Zhihao Zhao, Jiahui Guan, Jianzhi Wu, Minghan Chen, Yilin Guo, Peilin Xie, Ying-Chih Chiang

**Affiliations:** 1 Kobilka Institute of Innovative Drug Discovery, School of Medicine, The Chinese University of Hong Kong, Shenzhen, Shenzhen, China; 2 School of Science and Engineering, The Chinese University of Hong Kong, Shenzhen, Shenzhen, China; 3 Division of Applied Oral Sciences and Community Dental Care, Faculty of Dentistry, The University of Hong Kong, Hong Kong, Hong Kong SAR, China

**Keywords:** BBB, blood-brain barrier-penetrating peptides, ESM2, feature fusion, iFeatureOmega, peptide prediction, TabPFN

## Abstract

Predicting blood–brain barrier (BBB)-penetrating peptides remains critical for peptide-based central nervous system drug delivery, yet model performance depends strongly on data curation and feature representation. In this study, we constructed a benchmark dataset from publicly available resources by merging peptide records and removing duplicate sequences, resulting in 426 positive and 6,865 negative samples. Each peptide was encoded using fused representations that combine protein language model embeddings with physicochemical descriptors, yielding a 2,121-dimensional feature space. After variance filtering, standardization, and mutual-information-based feature selection, the top 700 features were retained for classification. To address class imbalance, the majority class in the training set was randomly undersampled to achieve a 1:5 positive-to-negative ratio. A foundation model for tabular classification, termed B3BPFN, was then trained on the processed feature matrix and evaluated on an independent balanced test set comprising 20% of the positive samples and an equal number of negative samples. The final model achieved a sensitivity of 0.9294, specificity of 0.8824, accuracy of 0.9059, Matthews correlation coefficient (MCC) of 0.8127, and area under the receiver operating characteristic curve (AUROC) of 0.9460. SHAP analysis further revealed that composition–transition–distribution (CTDD) descriptors serve as important features for BBB-penetrating peptide prediction. A user-friendly web server is freely available at https://ycclab.cuhk.edu.cn/b3bpfn to facilitate community use.

## Introduction

**FIGURE 1:**
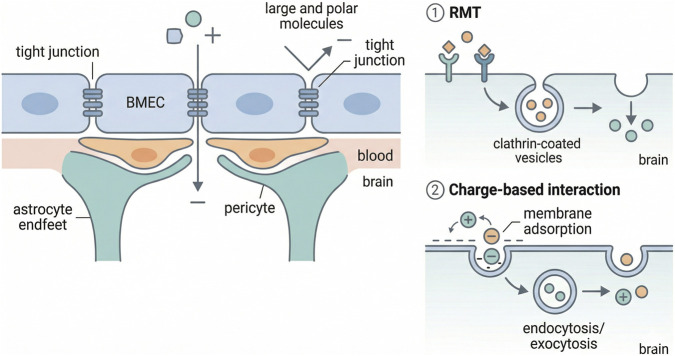
Schematic illustration of the blood–brain barrier (BBB) structure and major transport mechanisms. Tight junctions between brain microvascular endothelial cells (BMECs) restrict paracellular diffusion, preventing most large and polar molecules from entering the brain. Transport across the BBB occurs primarily through mechanisms such as receptor-mediated transcytosis (RMT) and charge-based adsorptive-mediated transcytosis, involving vesicular trafficking processes.

The blood–brain barrier (BBB) protects the brain, but it also makes treatment difficult. This specialized neurovascular interface is built from brain microvascular endothelial cells, pericytes, astrocyte endfeet, and a basement membrane. Tight junctions seal the endothelial layer, while low paracellular permeability and active transport systems restrict exchange across the barrier (Joan Abbott et al., 2010; Daneman and Prat, 2015; Sweeney et al., 2019). Together, these features maintain central nervous system homeostasis but block most therapeutic agents from entering the brain (Pardridge, 2005). BBB transport remains one of the central challenges in CNS drug delivery (Nance et al., 2021) (Figure 1).

BBB-penetrating peptides offer one way to cross this barrier. They can be linked to drugs, proteins, or nucleic acids, which makes them attractive delivery carriers (Parrasia et al., 2022; Kumar et al., 2021a; Dean et al., 2024). Yet discovery still relies on a slow experimental pipeline. Candidate sequences are synthesized one by one, tested in cell-based BBB assays or animal models, and only then judged for BBB penetration (He et al., 2014; Naik and Cucullo, 2012). This workflow is informative, but it is resource-intensive, time-consuming, and difficult to scale (Chen X. et al., 2022; Ma and Wolfinger, 2023). A recent meta-analysis further showed that BBB peptide shuttles are not the same as generic cell-penetrating peptides, because BBB penetration requires compatibility with the endothelial barrier rather than cellular uptake alone. They are usually small, hydrophobic, sparsely aromatic, and slightly cationic (Cavaco et al., 2024). This narrow profile makes sequence-based pre-screening especially useful and has motivated machine-learning approaches for early-stage filtering.

Curated resources such as B3Pdb collected experimentally validated BBB-penetrating peptides and supported model development (Kumar et al., 2021a). Early predictors such as BBPpredict and B3Pred relied on handcrafted sequence descriptors, feature selection, and standard classifiers (Chen X. et al., 2022; Kumar et al., 2021b). These studies showed that BBB-penetrating sequence patterns can be learned, but they also highlighted the limits of hand-designed features. Later work therefore focused on the main bottlenecks in this task: small sample size and class imbalance. Augur used borderline-SMOTE-based augmentation (Gu et al., 2024), DeepB3P3 used masked peptide transformers with dynamic routing (Ma and Wolfinger, 2023), and DeepB3P added feedback GAN augmentation (Tang and Chen, 2025). These methods improved prediction, but they still depended heavily on engineered features or extra augmentation, and sequence context was only partly captured by these approaches.

Despite these advances, a clear gap remains. A more direct capture of sequence context is still needed, while the final model must remain reliable on small, imbalanced data (Yu et al., 2015). Protein language models such as ESM2 can learn richer sequence patterns directly from amino acid context (Lin et al., 2023; Brandes et al., 2022), while iFeatureOmega adds interpretable physicochemical descriptors (Chen Z. et al., 2022). Given the compact feature space and the limited training data, TabPFN is well suited as the classifier (Hollmann et al., 2025). Accordingly, we combine ESM2 embeddings with physicochemical descriptors, followed by feature selection and class rebalancing, to build a compact predictor for BBB-penetrating peptides.

## Materials and methods

**FIGURE 2:**
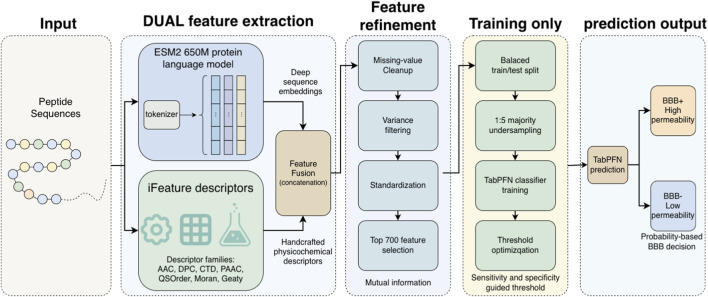
Overview of the proposed BBB-penetrating peptide prediction framework.Peptide sequences are first encoded using ESM2 to obtain contextual embeddings and iFeatureOmega to extract physicochemical descriptors. The two feature groups are then concatenated and processed through standardization and mutual-information-based feature selection. Finally, the selected features are fed into a pre-trained TabPFN classifier for prediction.

The overall workflow of the proposed method is illustrated in Figure 2.

### Dataset construction

Positive peptides were curated from B3Pdb, an experimentally validated database of BBB-penetrating peptides (Kumar et al., 2021a). Negative peptides were sampled from UniProt/Swiss-Prot (UniProt Consortium, 2023) using BBB-related exclusion keywords (e.g., blood brain barrier, brain, brainpeps, b3pdb, permeation, permeability, transmembrane, transport, transfer, membrane, neuro, and hemolysis), and duplicate sequences were removed from both classes. The final benchmark dataset contained 426 positive and 6865 negative peptides, with labels $y_{i}=1$ and $y_{i}=0$ , respectively.

Let $D={\left\{\left(s_{i},y_{i}\right)\right\}}_{i=1}^{N}$ , where $s_{i}$ denotes a peptide sequence and $y_{i}\in \left\{0,1\right\}$ denotes its class label. The cleaned dataset was split into training and test subsets as shown in Equation 1:

$$D=D_{\text{train}}\cup D_{\text{test}},\;D_{\text{train}}\cap D_{\text{test}}=\emptyset .$$ (1)

To obtain an independent balanced test set, 20% of the positive samples were reserved for testing, and the same number of negative samples were randomly selected. With a fixed random seed of 42, this split yielded a training pool of 341 positive and 6780 negative peptides, together with a balanced test set of 85 positive and 85 negative peptides. During classifier training, random undersampling further reduced the majority class to 1705 negative peptides, giving a rebalanced TabPFN reference set of 341 positive and 1705 negative peptides.

### Sequence representation

To capture both contextual and biochemical information, each peptide was represented by ESM2 embeddings and iFeatureOmega descriptors.

ESM2 first converted each sequence into a token sequence (Equation 2):

$$t_{i}=Tokenizer\left(s_{i}\right),$$ (2)

which was then mapped to an initial embedding matrix (Equation 3):

$$H_{i}^{\left(0\right)}=Embed\left(t_{i}\right).$$ (3)

The hidden states were updated layer by layer through the Transformer blocks of ESM2 (Equation 4):

$$H_{i}^{\left(\ell \right)}=\Phi^{\left(\ell \right)}\left(H_{i}^{\left(\ell -1\right)}\right),\;\ell =1,\ldots ,L,$$ (4)

where $\Phi^{\left(\ell \right)}$ denotes the ℓ -th Transformer block and L is the total number of layers. The final residue-level states were aggregated by masked sum pooling (Equation 5):

$$e_{i}=\overset{T_{i}}{\underset{j=1}{\sum }}m_{ij}H_{i,j}^{\left(L\right)},$$ (5)

where $T_{i}$ denotes the sequence length of peptide $s_{i}$ and $m_{ij}$ is the validity mask for residue j . The pretrained facebook/esm2_t33_650M_UR50D model was used in this study (Lin et al., 2023). The attention computation follows the standard Transformer formulation and is not expanded further here.

In parallel, iFeatureOmega was used to extract explicit physicochemical descriptors (Chen Z. et al., 2022). The descriptor set included amino acid composition (AAC) (Smith, 1966), dipeptide composition (DPC type 1) (Petrilli, 1993), composition–transition–distribution descriptors (CTDC, CTDT, CTDD) (Govindan and Nair, 2011), pseudo amino acid composition (PAAC) (Chou, 2009), quasi-sequence-order (QSOrder) (Chou, 2000), amphiphilic pseudo amino acid composition (APAAC) (Chou, 2005), grouped amino acid composition (GAAC) (Smith, 1966), Moran autocorrelation (Mathur, 2015), and Geary autocorrelation (Mathur, 2015). After concatenation and removal of duplicated descriptor columns, the resulting vector was written as Equation 6:

$$p_{i}=g_{\text{iFeatureOmega}}\left(s_{i}\right)\in R^{841}.$$ (6)

This representation encodes composition, local order, and autocorrelation features that complement the contextual information from ESM2.

### Feature fusion and selection

The two feature groups were concatenated to form a fused representation (Equation 7):

$$x_{i}=\left[e_{i};p_{i}\right]\in R^{2121}.$$ (7)

Before model training, invalid values were handled by the implementation defaults, and constant features were removed by variance filtering (Equation 8):

$$F_{\text{var}}=\left\{j|Var\left(x_{\cdot j}\right)>0\right\},\;{\tilde{x}}_{i}=x_{i}\left[F_{\text{var}}\right].$$ (8)

The filtered matrix was then standardized as Equation 9:

$$z_{i}=\frac{{\tilde{x}}_{i}-\mu }{\sigma },$$ (9)

where μ and σ were computed from the training set.

To reduce redundancy, mutual-information-based feature selection was applied (Amiri et al., 2011). For each standardized feature $z_{\cdot j}$ , the relevance score was defined as Equation 10:

$$I_{j}=I\;\left(z_{\cdot j};y\right),$$ (10)

and the top 700 features were retained according to Equation 11:

$$F_{700}=TopK\;\left(\left\{I_{j}\right\},700\right),\;u_{i}=z_{i}\left[F_{700}\right].$$ (11)

This stage compressed the fused representation into a more compact and discriminative space.

### TabPFN classification

Because negative samples greatly outnumbered positive samples, the training set was rebalanced by random undersampling of the majority class. After feature selection, the negative class was reduced to a positive:negative ratio of 1:5, which helped prevent the classifier from being dominated by the majority class (Equation 12):

$$|D_{\text{reb}}^{-}|=5\;|D_{\text{train}}^{+}|,$$ (12)

where $D_{\text{train}}^{+}$ denotes the positive training subset and $D_{\text{reb}}^{-}$ denotes the randomly undersampled negative subset.

TabPFN, short for Tabular Prior-data Fitted Network, is a transformer-based foundation model for tabular prediction (Hollmann et al., 2025). Its architecture assigns representations to table cells and employs row-wise and column-wise attention, first across features within each sample and then across samples for the same feature. Unlike conventional classifiers that are trained separately on each dataset, TabPFN is pre-trained offline on millions of synthetic tabular tasks. During pretraining, a synthetic task T is split into a labeled context set and a masked target set, and the model is optimized to recover the masked labels using the task loss in Equation 13 and the pretraining objective in Equation 14:

$$L_{T}\left(\theta \right)=\underset{j\in M_{T}}{\sum }\ell \;\left(f_{\theta }\left(x_{j},T_{\text{ctx}}\right),y_{j}\right),$$ (13)

$$\theta^{*}=\arg\min_{\theta }E_{T∼p\left(T\right)}\left[L_{T}\left(\theta \right)\right],$$ (14)

where $T_{\text{ctx}}$ is the context set and $M_{T}$ is the set of masked targets. This training strategy teaches the model a generic inference rule for tabular data rather than a dataset-specific decision boundary.

For each peptide, the selected feature vector was evaluated by a pre-trained TabPFN classifier using the rebalanced training set as in-context reference, and the resulting output score was interpreted as the predicted probability of the positive class (Equation 15):

$${\hat{p}}_{i}=f_{\theta }\left(U_{\text{train}},y_{\text{train}},u_{i}\right),$$ (15)

where $f_{\theta }$ denotes the pre-trained TabPFN predictor. The final label was then obtained by thresholding (Equation 16):

$${\hat{y}}_{i}=1\left({\hat{p}}_{i}\geq \tau \right),$$ (16)

where τ denotes the decision threshold. For the final reported operating point, the stored threshold in the saved model package was τ=0.215 ; this same threshold was used for held-out test evaluation and literature-derived candidate inference. In our implementation, TabPFN was used in its standard pre-trained inference mode, while the task-specific training data were supplied through the fit procedure as contextual reference rather than through dataset-specific gradient-based fine-tuning of model weights. The complete modeling chain therefore consisted of ESM2 embedding, physicochemical feature extraction, feature fusion, feature selection, class rebalancing, and TabPFN classification.

### Performance evaluation

To evaluate prediction performance, the confusion-matrix counts—true positives (TP), true negatives (TN), false positives (FP), and false negatives (FN)—were used to compute sensitivity, specificity, accuracy, precision, F1 score, and MCC, respectively (Equations 17–22):

$$Sn=\frac{TP}{TP+FN},$$ (17)

$$Sp=\frac{TN}{TN+FP},$$ (18)

$$ACC=\frac{TP+TN}{TP+TN+FP+FN},$$ (19)

$$Precision=\frac{TP}{TP+FP},$$ (20)

$$F1=\frac{2\cdot Precision\cdot Sn}{Precision+Sn},$$ (21)

$$MCC=\frac{TP\cdot TN-FP\cdot FN}{\sqrt{\left(TP+FP\right)\left(TP+FN\right)\left(TN+FP\right)\left(TN+FN\right)}}.$$ (22)

In addition, the area under the receiver operating characteristic curve (AUROC) and the area under the precision–recall curve (AUPRC) were computed by integrating the respective curves using the trapezoidal rule. AUROC summarizes ranking performance across classification thresholds, whereas AUPRC is more informative when class imbalance is severe (McDermott et al., 2024). Together, these metrics provide complementary views of screening quality for BBB-penetrating peptide prediction.

## Results

### Baseline comparison

Baseline performance was first assessed across three input representations: iFeature descriptors alone, ESM2 embeddings alone, and their fusion.

iFeature descriptors alone yielded an MCC of 0.7791 and an ACC of 0.8882, indicating that physicochemical summaries already retain strong signal for BBB peptide prediction (Supplementary Table S2). Using ESM2 embeddings alone reduced MCC to 0.7011 and ACC to 0.8471, suggesting that contextual embeddings by themselves do not fully replace the discriminative information captured by handcrafted peptide descriptors in this benchmark.

Naive fusion of the two representations without subsequent feature selection produced an MCC of 0.7430 and an ACC of 0.8706, indicating that simple dimensional expansion alone was not sufficient to outperform the stronger iFeature-only baseline. This pattern suggests that the descriptor set and the language-model embedding still contribute complementary information, but that the fused space must be compressed to suppress redundant or noisy dimensions before the combined signal becomes fully useful.

Feature selection on the fused representation revealed a clear optimum. In a top-*k* sweep, MCC increased from 0.743 with the full 2121-dimensional fused matrix to 0.777 (top 100), 0.788 (top 300), 0.788 (top 500), and 0.8127 (top 700), before decreasing to 0.765 (top 1000) and 0.718 (top 1500). The maximum at 700 features indicates that the fused representation is most effective after redundant dimensions are removed and the retained signal is concentrated in a compact informative subspace.

Based on this result, the top-700 fused feature set was adopted as the final representation.

### Comparison with existing models

Performance on the same held-out test set was then compared with that of previously reported BBB-penetrating peptide predictors. Full results for all methods are summarized in Table 1.

**TABLE 1:** Performance comparison of the proposed BBB-penetrating peptide prediction model and existing predictors on the held-out test set. MCC is used as the primary discrimination metric. All methods were trained and evaluated on the same split used for the final model. Among the compared baselines, deepB3P and DeepB3P3 were evaluated using publicly available implementations, whereas Augur, BBPpredict, and B3Pred were reproduced from the methodological details reported in their original publications; further implementation details are provided in the Supplementary Notes on Comparative Models. Bold values indicate the best result in each column.

Model	AUROC	Sensitivity (Sn)	Specificity (Sp)	Accuracy (ACC)	MCC
Final (fused ESM2 + iFeatureOmega + TabPFN)	**0.9460**	**0.9294**	0.8824	**0.9059**	**0.8127**
B3Pred	0.8761	0.6706	0.9176	0.7941	0.6071
deepB3P	0.8865	0.6235	0.9412	0.7824	0.5956
BBPpredict	0.9230	0.3176	1.0000	0.6588	0.4345
DeepB3P3	0.7912	0.3007	1.0000	0.6504	0.4207
Augur	0.8531	0.2353	1.0000	0.6176	0.3651

The final framework achieved an AUROC of 0.9460, Sn of 0.9294, Sp of 0.8824, ACC of 0.9059, and MCC of 0.8127, outperforming all competing methods on every reported metric. At the stored threshold of 0.215, the corresponding confusion-matrix counts were TP = 79, FN = 6, TN = 75, and FP = 10. Among the alternatives, B3Pred and deepB3P showed the most balanced sensitivity–specificity trade-offs, but their MCC values remained lower at 0.6071 and 0.5956, with sensitivities of 0.6706 and 0.6235, respectively. A different pattern emerged for BBPpredict, DeepB3P3, and Augur: all three reached perfect specificity on the test set, but only by sacrificing sensitivity to 0.3176, 0.3007, and 0.2353, respectively. BBPpredict illustrates this limitation clearly, because its AUROC of 0.9230 still coincides with sensitivity low enough to discard most true positives at the initial screening stage.

The final model maintained the highest sensitivity among all methods while preserving competitive specificity. This balance is reflected in its MCC of 0.8127, which exceeds the strongest competing value of 0.6071 by a substantial margin and indicates that the gain extends across the full confusion matrix rather than favoring only one error type.

### Visualization analysis

**FIGURE 3:**
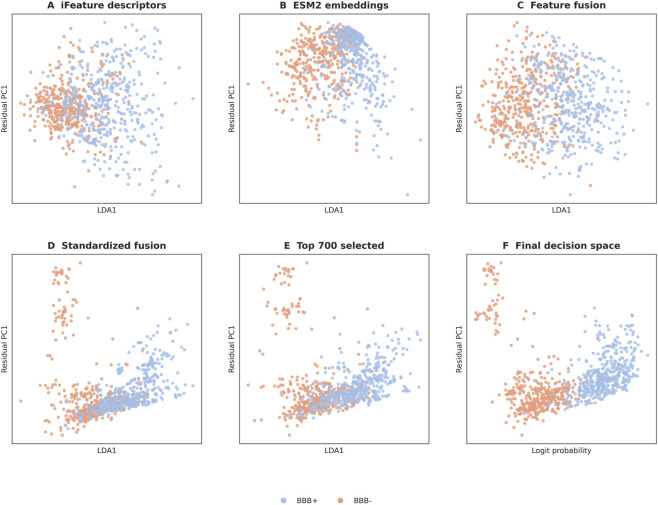
Two-dimensional projections of the learned representations at successive pipeline stages. **(A)** iFeature descriptors only. **(B)** ESM2 embeddings only. **(C)** Fused ESM2 + iFeatureOmega representation. **(D)** After standardization of the fused features. **(E)** After top-700 feature selection. **(F)** Final logit-probability score space. Blue: BBB-penetrating peptides (BBB+); orange: non-BBB-penetrating peptides (BBB-).

A geometric view of the pipeline was obtained by projecting the learned representations into two dimensions at each major stage (Figure 3), allowing the numerical gains to be compared with visible changes in feature-space organization.

In the iFeature-only representation (Panel A), the BBB+ and BBB- classes overlap substantially. Replacing descriptors with ESM2 embeddings (Panel B) reduces this overlap, and fusing the two representations (Panel C) sharpens the separation further. Standardization of the fused features (Panel D) produces a more uniform spread by removing scale-driven distortion, whereas top-700 feature selection (Panel E) contracts the class clusters by discarding dimensions that do not improve class resolution. In the final score space (Panel F), the two classes are separated with minimal overlap, consistent with the MCC of 0.8127 reported in Table 1.

**FIGURE 4:**
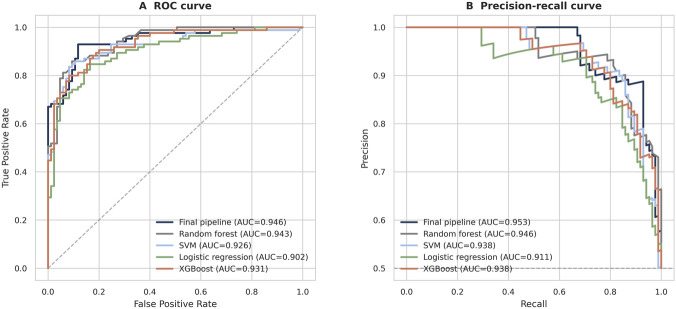
**(A)** ROC curves for the final pipeline and alternative classifiers on the held-out test set. **(B)** Precision-recall curves for the final pipeline and alternative classifiers on the held-out test set.

Figure 4 provides a threshold-independent view of the same progression. The final pipeline maintains the strongest ROC and precision–recall behavior across the compared classifiers, showing that the advantage is not confined to a single operating point but persists across a broad range of decision thresholds.

### Ablation and feature selection

The contributions of the main pipeline components were examined through three groups of ablation experiments: feature-subset sweeps, component removal, and classifier replacement.

**FIGURE 5:**
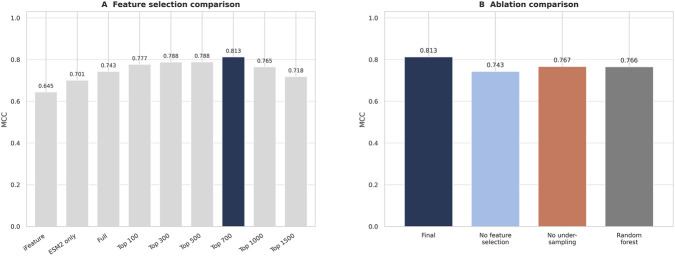
**(A)** MCC as a function of the number of selected features; the top-700 subset yields the highest value. **(B)** MCC after removal or replacement of individual pipeline components.

In the feature-subset sweep, the top-700 configuration yielded the highest MCC of 0.8127. Smaller subsets retained much of the signal but did not recover the full gain, with MCC values of 0.777 (top 100) and 0.788 (top 300 and top 500). Larger subsets reversed this trend, with MCC decreasing to 0.765 at 1000 features and 0.718 at 1500 features (Figure 5).

Component-removal experiments assessed the contributions of feature selection and class rebalancing independently. Eliminating feature selection reduced MCC from 0.8127 to 0.743 while AUROC remained at 0.946, indicating that the full fused representation preserves ranking ability but supports a less precise decision boundary. Removing majority-class undersampling lowered MCC to 0.767 and shifted the classifier toward lower sensitivity on the minority class (Supplementary Table S3).

Classifier replacement was tested by pairing the same top-700 feature matrix with random forest, SVM, XGBoost, and logistic regression. MCC decreased to 0.766, 0.756, 0.723, and 0.639, respectively (Supplementary Table S3). Because all classifiers operated on the same processed feature set, these differences reflect classifier-level rather than representation-level effects.

As a supplementary stability check, five-fold stratified cross-validation on the training pool using the same preprocessing and modeling strategy yielded a mean AUROC of 0.921 ± 0.011 (Supplementary Table S4). Because all folds originated from the same curated source, this result reflects internal consistency across different train–validation partitions and repeated undersampling rather than external validation.

### Prediction on literature-derived external candidates

As a preliminary external validation beyond the reconstructed benchmark, a small literature-derived set of four experimentally reported BBB-related peptides was assembled. None of these candidates showed an exact sequence match to either the positive or negative pools used in this study. All four peptides were excluded from model development, and inference was performed using the frozen final pipeline with the same decision threshold (τ=0.215) applied in the held-out test evaluation.

All four candidates were classified as BBB-penetrating by the final model. M1 (TFYGGRPKRNNFLRGIR) received the highest probability score (0.9556), followed by PB5-3 (QFAALPVRAHYG; 0.4489), NFL-TBS.40-63 (YSSYSAPVSSSLSVRRSYSSSSGS; 0.2486), and RAP12 (EAKIEKHNHYQK; 0.2377). Although RAP12 and NFL-TBS.40-63 scored only modestly above the operating threshold, none of the externally curated candidates was rejected. This result is consistent with the intended role of the framework as a sensitivity-preserving screening tool that prioritizes plausible BBB-penetrating peptides for downstream experimental follow-up, rather than imposing an overly conservative filter at the initial screening stage. Candidate identities, evidence tiers, and source PMIDs are summarized in Supplementary Table S7.

### Interpretability analysis

**FIGURE 6:**
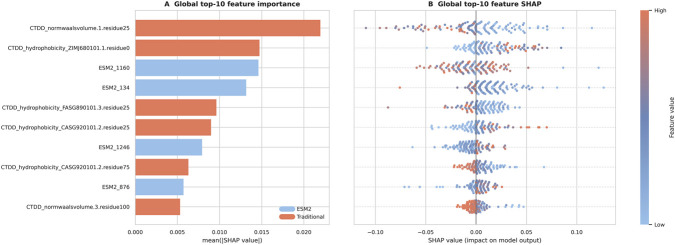
SHAP-based feature importance for the final TabPFN model. **(A)** Top 10 features ranked by mean absolute SHAP value. Six belong to the CTDD descriptor family (hydrophobicity- and normalized van der Waals volume-related); four are ESM2 embedding dimensions. **(B)** Beeswarm plot showing the distribution of SHAP values across all test samples. Each point represents one sample; color indicates feature value (red: high; blue: low). SHAP values indicate feature influence on model predictions and do not by themselves establish BBB transport mechanisms, receptor specificity, or causal determinants.

SHAP values computed for the TabPFN model trained on the top-700 feature set were used to characterize which features drive the final predictions (Figure 6).

Among the 10 features with the largest mean absolute SHAP values, six belonged to the CTDD descriptor family and four were ESM2 embedding dimensions. The CTDD features with the highest attribution scores were related to hydrophobicity and normalized van der Waals volume, properties previously associated with BBB-penetrating peptide behavior (Cavaco et al., 2024). These descriptors are biologically plausible because hydrophobicity and residue volume influence peptide compactness, residue packing, and the balance between polar and nonpolar surface character. Such a constrained polarity–hydrophobicity balance is consistent with reported BBB shuttle properties, in which peptides often need sufficient membrane compatibility without becoming broadly nonspecific cell-penetrating sequences (Cavaco et al., 2024). In the SHAP beeswarm plot (Figure 6B), these CTDD features showed clear directional effects on model output, with higher or lower feature values consistently pushing predictions toward one class. The four ESM2 dimensions displayed more dispersed and symmetric attribution patterns across samples, indicating context-dependent rather than monotonic contributions. These attribution patterns identify features that influenced model predictions but should not be interpreted as direct evidence of transport mechanism, receptor specificity, or causal determinants of BBB crossing.

Supplementary permutation-based analysis at the descriptor-family level was consistent with these observations (Supplementary Tables S5, S6). CTDD was the strongest individual handcrafted family, while the retained ESM2 dimensions collectively produced the largest overall MCC drop when permuted. The final model therefore draws on both interpretable physicochemical descriptors and contextual embedding features, and neither source alone accounts for the full predictive signal.

### Web interface

For practical use, a public web server named B3BPFN was developed and is freely available at https://ycclab.cuhk.edu.cn/b3bpfn/.

The server accepts peptide sequences in plain-text format (one sequence per line) or standard FASTA format with >ID headers. Each submission is limited to 50 sequences, and each sequence may contain no more than 512 residues drawn from the 20 canonical amino acids (ACDEFGHIKLMNPQRSTVWY). A FASTA file upload option is also provided, with a size limit of 1 MB.

**FIGURE 7:**
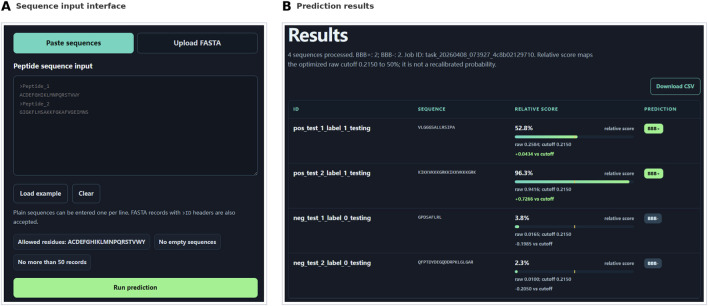
**(A)** Sequence input interface of the B3BPFN server. **(B)** Prediction results returned by the server, including BBB+/BBB- classification and predicted probability for each query sequence.

Upon submission, the server executes the full B3BPFN pipeline on the back end, from ESM2 encoding and iFeatureOmega descriptor extraction through feature fusion, selection, and TabPFN classification. For each query peptide, it returns a BBB + or BBB − prediction together with the associated probability score. Results are displayed directly on the output page after computation is complete. The web server interface is illustrated in Figure 7. The benchmark dataset and the source code for the prediction pipeline are also available at the same URL.

## Discussion

The results of this study demonstrate that accurate prediction of BBB-penetrating peptides benefits from combining complementary sources of sequence information and that the observed gains arise from a cumulative refinement process rather than any single modeling choice.

Traditional physicochemical descriptors based on composition, transition, distribution, and autocorrelation encode broad sequence properties such as hydrophobicity, residue volume, and local residue ordering patterns. These statistics are interpretable and biologically grounded, but they reduce each peptide to predefined global summaries, so peptides with similar overall chemistry but different residue arrangements can remain difficult to separate. ESM2 embeddings, by contrast, preserve contextual and positional information learned from large-scale protein corpora (Lin et al., 2023). For BBB penetration, such context-sensitive encoding is biologically plausible because transport-related behavior is unlikely to depend solely on averaged physicochemical composition; residue spacing, local motifs, and higher-order sequence organization are also expected to contribute (Joan Abbott et al., 2010; Daneman and Prat, 2015; Sweeney et al., 2019; Cavaco et al., 2024). In the present benchmark, the iFeature-only baseline remained stronger than the ESM2-only baseline, indicating that handcrafted physicochemical summaries still capture a large fraction of the discriminative signal. At the same time, the top-700 fused representation ultimately surpassed both single-family models, showing that the two feature families do capture non-redundant aspects of sequence information once the expanded fused space is pruned into a compact informative subspace. The gain from fusion therefore reflects complementarity revealed by feature selection rather than by dimensional expansion alone.

Although the full 2121-dimensional fused representation already achieved reasonable ranking performance (AUROC 0.946), selecting the top 700 features by mutual information raised MCC from 0.743 to 0.8127. The peak at 700 features, followed by a decline at larger subsets, indicates that the key improvement came from enriching the representation and then concentrating it into a compact informative subspace; additional dimensions beyond this point introduced more redundancy than discriminative signal, destabilizing the decision boundary. Removing majority-class undersampling led to a distinct failure mode: the model became more conservative toward the minority class, reducing sensitivity even when overall ranking remained strong. In an imbalanced screening setting where false negatives are lost before any wet-lab validation begins while false positives can still be filtered in later assays, such a shift would substantially reduce the preservation of plausible positive candidates.

Replacing TabPFN with random forest, SVM, XGBoost, or logistic regression on the same top-700 feature set consistently lowered MCC. These comparisons do not imply that TabPFN is universally superior to the alternatives; rather, they suggest that its inductive bias, a prior learned from synthetic tabular tasks, is particularly well aligned with the present problem, namely, a compact and heterogeneous feature matrix derived from limited positive data. Because all classifiers operated on the same processed feature set, the performance differences cannot be attributed to feature engineering alone and instead reflect how each classifier exploits the structure of the input representation.

The stage-wise projections in Figure 3 provide geometric confirmation of the numerical gains. Standardization removed distortion caused by heterogeneous feature scales, preventing a small number of large-magnitude dimensions from dominating the geometry for numerical rather than biological reasons. Top-700 selection then contracted the class clusters by removing dimensions that added width without improving class resolution. The progressive tightening of the overlap region across pipeline stages shows that the classifier is not rescuing a weak representation; it is exploiting a feature space that has already been substantially refined by contextual encoding, fusion, scale correction, and dimensional pruning. The threshold-independent ROC and precision–recall curves (Figure 4) reinforce this conclusion by showing that the advantage persists across a broad range of operating points rather than being confined to one convenient decision threshold.

The final model achieved the highest MCC (0.8127) among all compared methods, substantially exceeding the closest alternatives (B3Pred: 0.6071; deepB3P: 0.5956). The remaining methods, BBPpredict, DeepB3P3, and Augur, achieved perfect specificity but at the cost of severely reduced sensitivity (as low as 0.2353), a pattern that effectively eliminates most BBB-penetrating candidates before experimental follow-up. For a discovery-oriented screening task, such conservatism is difficult to justify operationally, because false positives can still be filtered in later assays whereas false negatives are permanently lost at the initial screen. The MCC margin therefore represents a genuine shift in screening behavior rather than a marginal score increment.

SHAP analysis revealed that the final model relies on a combination of CTDD descriptors and ESM2 embedding dimensions, with six of the top ten features belonging to the CTDD family. The prominence of hydrophobicity-related descriptors and normalized van der Waals volume terms is consistent with current understanding of BBB-relevant peptide properties, in which penetration is shaped by a constrained balance of polarity, compactness, residue packing, and surface chemistry (Joan Abbott et al., 2010; Daneman and Prat, 2015; Sweeney et al., 2019; Cavaco et al., 2024). The ESM2 dimensions contributed at a different level: their SHAP effects were more dispersed and context-dependent than those of the CTDD descriptors, consistent with a representation that captures distributed sequence relationships rather than simple monotonic trends (Lin et al., 2023). The descriptor features therefore provide the clearer physicochemical backbone of the prediction, whereas the embedding features encode subtler aspects of sequence organization whose predictive value depends on surrounding context. It should be noted, however, that SHAP values and permutation importance identify statistically influential features but do not establish transport mechanisms or receptor specificity. The interpretability claimed here is therefore at the level of biologically plausible and internally consistent feature attribution, which is appropriate for candidate prioritization but does not substitute for mechanistic validation.

Several limitations should be acknowledged. The dataset was reconstructed from released data files and remains relatively small on the positive class, which may limit generalization to newly discovered peptide families. The five-fold cross-validation result (mean AUROC 0.921 ± 0.011) confirmed internal consistency of the pipeline across different train–validation partitions and repeated undersampling, but all folds originated from the same curated source and therefore do not constitute external validation. Similarly, the four literature-derived candidates provide only a small sanity check under the frozen pipeline and should not be interpreted as broad external validation. Furthermore, the current framework focuses exclusively on sequence-level prediction and does not incorporate experimentally measured transport mechanisms, structural conformations, or receptor-specific interactions. As training data grow, the scalability of TabPFN’s in-context learning paradigm may also become a concern, and alternative classifiers could become more competitive. In addition, the current model provides binary classification rather than a quantitative estimate of penetration efficiency, which limits its utility for ranking candidates by expected transport rate. Future work may benefit from integrating structural modeling or mechanism-aware annotations, expanding the training set with independently curated peptide collections, adopting regression-based formulations where graded activity data are available, and conducting prospective experimental validation to assess real-world screening utility.

## Conclusion

This study presented B3BPFN, a sequence-based framework for predicting blood–brain barrier-penetrating peptides that combines ESM2 protein language model embeddings with iFeatureOmega physicochemical descriptors under a TabPFN classifier. Fusion of the two complementary representations, followed by variance filtering and mutual-information-based selection, produced a compact 700-dimensional feature space that achieved a sensitivity of 0.9294, specificity of 0.8824, accuracy of 0.9059, MCC of 0.8127, and AUROC of 0.9460 on an independent test set, exceeding all compared methods in overall discrimination while maintaining the most balanced sensitivity–specificity profile among them.

Ablation experiments confirmed that the performance gain arises from the alignment of multiple pipeline components, including feature fusion, dimensional compression, class rebalancing, and classifier selection, rather than from any single design choice. Interpretability analysis further showed that the model draws on biologically plausible physicochemical properties alongside contextual embedding features, supporting confidence in the relevance of the learned signal. A publicly available web server at https://ycclab.cuhk.edu.cn/b3bpfn/provides direct access to the full prediction pipeline.

Key limitations include the small size of the positive class in the current benchmark and the absence of external validation on independently curated datasets. Future work incorporating structural information, graded permeability measurements, and prospective experimental testing could further strengthen the framework. Despite these open directions, the present results demonstrate that fusing language-model and physicochemical representations under a compact feature-selection and foundation-model classification pipeline offers a practical and interpretable approach to BBB-penetrating peptide prediction.

## Data Availability

The datasets and trained model checkpoints used in this study are publicly available at https://github.com/Carsonn-Liu/B3BPFN.
