# Supplementary material for: Prediction of blood-brain barrier-penetrating peptides using B3BPFN
Source: Front Mol Biosci. 2026 May 20;13:1858506. doi: 10.3389/fmolb.2026.1858506 (PMC13229726; doi:10.3389/fmolb.2026.1858506)
Supplement: Supplementary file 1 [file DataSheet1.pdf]

Supplementary Information

Prediction of Blood-Brain Barrier-Penetrating Peptides Using Fused ESM2 Embeddings and Physicochemical Descriptors

1 Supplementary Methods

1.1 Dataset provenance and cleaning summary

The benchmark dataset used in the main manuscript was reconstructed from publicly available peptide resources. Positive samples were curated from B3Pdb, whereas negative samples were collected from UniProt/Swiss-Prot after excluding BBB-related entries by keyword filtering. Duplicate sequences were removed within and across classes before model development. After cleaning, the final dataset contained 426 BBB-penetrating peptides and 6865 non-BBB-penetrating peptides.

The same deterministic split described in the main manuscript was used throughout the repository. With a fixed random seed of 42, 20% of the positive set was held out for testing, and an equal number of negative samples was drawn to form a balanced test set. This yielded 341 positive and 6780 negative samples for the original training pool, together with 85 positive and 85 negative samples for testing. During classifier training, the majority class in the training pool was randomly undersampled to a 1:5 positive-to-negative ratio, resulting in a rebalanced training subset of 341 positive and 1705 negative samples.

1.2 Feature construction and model configuration

Each peptide was represented by the concatenation of two feature families. First, contextual sequence embeddings were extracted from the pretrained `facebook/esm2_t33_650M_UR50D` protein language model, yielding a 1280-dimensional sequence representation after masked sum pooling. Second, iFeatureOmega descriptors were computed from the same peptide sequence, yielding 841 physicochemical descriptors after concatenation and duplicate-column removal. The fused feature representation therefore had 2121 dimensions before preprocessing.

The preprocessing pipeline matched the saved final-model artifacts used in the main study. Constant features were removed by variance filtering, the remaining columns were standardized, and mutual-information-based feature selection was used to retain the top 700 features. A pretrained TabPFN classifier was then fitted on the rebalanced training subset. For the final-pipeline rows reported here, the operating point matches that used in the main manuscript, namely the threshold of 0.215 retained in the saved model package and recorded in the final training log.

1.3 Cross-validation protocol

In addition to the independent balanced test set reported in the main manuscript, a supplementary 5-fold stratified cross-validation analysis was carried out on the original training pool of 7121 peptides (341 positive and 6780 negative). For each fold, variance filtering, standardization, mutual-information-based top-700 feature selection, majority-class undersampling to a 1:5 ratio, and TabPFN fitting were repeated using only the fitting split of that fold. The held-out validation fold was kept at its original class distribution and was not rebalanced. Because

these validation folds remained strongly imbalanced, cross-validation stability is summarized primarily with the threshold-independent AUROC metric in Table S4.

1.4 Global feature-attribution analysis

To further interpret the final fused model, a supplementary global feature-attribution analysis was performed on the 170-peptide independent test set. First, permutation-based importance scores were used to rank the 700 selected features. These feature-level results were then aggregated by descriptor family to summarize the relative contribution of each retained feature family. Conditional-permutation SHAP values were subsequently computed for the 10 highest-ranked features to summarize their global contribution magnitudes. The resulting supplementary tables therefore provide both a family-level view and an individual-feature view of the final model, rather than an exhaustive analysis of the full 2121-dimensional preselection space.

1.5 Sources reused for this supplement

No new training or embedding extraction was performed for this supplement. All numerical summaries reported below were derived from precomputed outputs generated during the main analysis workflow:

- the saved final-training log for the main-model metrics reported in the manuscript;
- cached feature-family and top-k comparison outputs for the expanded representation analysis;
- cached ablation, classifier-swap, and source-data outputs for the supplementary performance summaries;
- a cached fused-feature matrix used to run the supplementary cross-validation analysis without recomputing ESM2 embeddings;
- cached global permutation-importance and conditional-permutation SHAP summaries for the final model; and
- benchmark summaries used for the implementation notes and parameter counts of comparative models.

2 Supplementary Results

Table S1: Dataset composition, deterministic split, and class rebalancing used by the final pipeline.

Stage or subset	Positive	Negative
Cleaned full dataset	426	6865
Original training pool	341	6780
Balanced test set	85	85
Rebalanced training subset used by TabPFN	341	1705

Table S2: Expanded feature-representation and top-k feature-selection results. The final operating point reported in the main manuscript corresponds to the fused representation with the top 700 features. All single-family and fused-model entries were evaluated under the same aligned retraining protocol and decision-threshold selection strategy.

Category	Configuration	Features	Threshold	Sn	Sp	ACC	MCC	AUROC
Single family	iFeature only	841	0.220	0.847	0.929	0.888	0.779	0.952
Single family	ESM2 only	1280	0.300	0.776	0.918	0.847	0.701	0.929
Fused representation	Full feature set	2121	0.275	0.835	0.906	0.871	0.743	0.946
Fused representation	Top 100	100	0.140	0.906	0.871	0.888	0.777	0.946
Fused representation	Top 300	300	0.195	0.894	0.894	0.894	0.788	0.947
Fused representation	Top 500	500	0.180	0.906	0.882	0.894	0.788	0.945
Fused representation	Top 700 (final)	700	0.215	0.929	0.882	0.906	0.813	0.946
Fused representation	Top 1000	1000	0.185	0.894	0.871	0.882	0.765	0.944
Fused representation	Top 1500	1500	0.210	0.835	0.882	0.859	0.718	0.939

Table S3: Expanded ablation and classifier-replacement results derived from cached prediction scores. The final pipeline uses the same threshold and performance values reported in the main manuscript.

Group	Variant	Threshold	Sn	Sp	ACC	MCC	AUROC
Ablation	Final pipeline	0.215	0.929	0.882	0.906	0.813	0.946
Ablation	Without feature selection	0.275	0.835	0.906	0.871	0.743	0.946
Ablation	Without undersampling	0.070	0.847	0.918	0.882	0.767	0.946
Classifier swap	Random forest	0.260	0.859	0.906	0.882	0.766	0.943
Classifier swap	SVM	0.155	0.835	0.918	0.876	0.756	0.926
Classifier swap	Logistic regression	0.225	0.765	0.871	0.818	0.639	0.902
Classifier swap	XGBoost	0.135	0.800	0.918	0.859	0.723	0.931

Table S4: Five-fold stratified cross-validation AUROC on the original training pool. Each fold repeated the same preprocessing and training sequence as the final pipeline, with majority-class undersampling applied only within the fitting split.

Fold	AUROC
1	0.904
2	0.931
3	0.925
4	0.927
5	0.921
Mean ± SD	0.921 ± 0.011

Table S5: Top 10 globally important features in the final model ranked by mean absolute SHAP value after conditional-permutation analysis.

Rank	Feature	Source	Descriptor family	Mean |SHAP|
1	CTDD_normwaalsvolume.1.residue25	Traditional	CTDD	0.0219
2	CTDD_hydrophobicity_ZIMJ680101.1.residue0	Traditional	CTDD	0.0147
3	ESM2_1160	ESM2	ESM2	0.0146
4	ESM2_134	ESM2	ESM2	0.0131
5	CTDD_hydrophobicity_FASG890101.3.residue25	Traditional	CTDD	0.0096
6	CTDD_hydrophobicity_CASG920101.2.residue25	Traditional	CTDD	0.0090
7	ESM2_1246	ESM2	ESM2	0.0079
8	CTDD_hydrophobicity_CASG920101.2.residue75	Traditional	CTDD	0.0063
9	ESM2_876	ESM2	ESM2	0.0057
10	CTDD_normwaalsvolume.3.residue100	Traditional	CTDD	0.0053

Table S6: Descriptor-family aggregation of permutation importance across the 700 selected features retained in the final model. Aggregated drops are reported as sums over all selected features within each family and are intended for relative comparison only.

Source	Descriptor family	Summed AUC drop	Summed MCC drop
Traditional	CTDD	0.0277	2.7424
Traditional	Moran	0.0018	0.0642
Traditional	DPC	0.0004	0.0162
Traditional	Geary	0.0001	0.0041
Traditional	PAAC	-0.0002	0.0504
Traditional	GAAC	-0.0004	0.0993
Traditional	CTDT	-0.0006	0.4755
Traditional	CTDC	-0.0008	0.5277
Traditional	AAC	-0.0012	0.2241
Traditional	QSOrder	-0.0014	0.1915
Traditional	APAAC	-0.0021	0.1520
ESM2	ESM2	-0.0064	4.8319

2.1 Literature-derived external candidate screen

To provide a small-scale literature-based external check, four BBB-related peptides were collected from primary studies after applying two filters. First, each candidate had to be described in the source article as having experimentally supported BBB, BBTB, or BBB-model penetration behavior. Second, the peptide sequence had to be absent by exact sequence match from both the positive and negative pools used in this study. The resulting set included two entries with direct in vivo or systemic-delivery support (RAP12 and PB5-3) and two additional exploratory entries supported by BBB/BBTB penetration experiments in tumor-focused or in vitro settings (M1 and NFL-TBS.40-63).

These four peptides were evaluated with the saved final pipeline without retraining. Inference used the same serialized preprocessing objects, TabPFN classifier, and fixed decision threshold of 0.215 described in the main manuscript. The resulting probabilities and binary labels are reported in Table S7.

Taken together, Tables S2, S3, S4, S5, S6, and S7 support the main results of the study. Under the aligned retraining protocol, the iFeature-only baseline already provided strong discrimination, whereas the ESM2-only representation performed somewhat less well when used without physicochemical descriptors. The top-700 fused representation yielded the best overall MCC, indicating that the main gain came from combining the two feature families and then removing redundant dimensions. Likewise, both informative feature selection and majority-class undersampling contributed materially to the final classification quality, whereas replacing

TabPFN with conventional classifiers led to lower MCC values on the same held-out test set. The cross-validation analysis further indicated that the same modeling strategy remained stable across training folds, with a mean AUROC of 0.921 ± 0.011 despite the marked class imbalance retained in the validation folds. The global attribution analysis further suggested that the final predictor drew on both learned ESM2 dimensions and traditional descriptors, with six of the 10 highest-ranked SHAP features originating from CTDD descriptors and four from ESM2 embeddings. At the descriptor-family level, CTDD provided the strongest aggregated contribution among the traditional descriptors, whereas the retained ESM2 dimensions also contributed substantial importance to the final decision function. In addition, the external candidate screen showed that all four literature-derived peptides exceeded the fixed decision threshold under the frozen final pipeline, which is consistent with the model's intended use as a sensitivity-oriented early screening tool.

3 Supplementary Reproducibility Notes

All primary results reported here were generated with a fixed random seed of 42 in the final training and figure-generation workflow. The accompanying code base contains dedicated scripts for final-model training, inference with the saved pipeline, baseline benchmarking, and figure generation.

The saved final-model package includes the variance selector, standard scaler, top-700 feature selector, trained TabPFN estimator, and the stored decision threshold. In addition, a cached fused-feature matrix covering all 7291 peptides was retained for figure generation and downstream benchmarking, together with auxiliary cached outputs for layer-wise embedding analysis and publication figures.

All compute jobs in the original analysis were managed through Slurm-based submission scripts on institutional GPU resources. The supplementary cross-validation analysis reused the cached fused-feature matrix and therefore did not require recomputation of protein language model embeddings.

4 Supplementary Notes on Comparative Models

The accompanying code base also includes local implementations or wrapped reproductions of five comparative BBB-penetrating peptide predictors. Their outputs were consolidated during the main benchmarking workflow.

- **B3Pred** was reproduced as a local implementation of the published 9189-dimensional composition-descriptor family, followed by SVC-L1 screening, LightGBM feature ranking, and a random-forest predictor trained on the selected top 80 features.
- **BBPpredict** was reproduced with AAC, DPC, GAAC, CKSAAGP, and PAAC descriptors, followed by F-score-based feature selection and a 63-tree random forest.
- **deepB3P** retained the public repository workflow, including FBGAN-based pseudo-positive generation and the published checkpoint structure; the reproduced model had approximately 7.07 million trainable parameters.
- **DeepB3P3** was benchmarked from a saved masked-peptide transformer checkpoint trained with 8× masked augmentation for 50 epochs; the reproduced model had approximately 2.87 million trainable parameters.
- **Augur** was reproduced with the released descriptor family, BorderlineSMOTE, random undersampling, mutual-information-based feature selection, and a random-forest classifier.

For reference, the final model had an estimated parameter count of approximately 661.76 million when the ESM2 backbone and tabular prediction stage were considered together. A separate fairness-oriented evaluation setting was generated during internal benchmarking, but this supplementary document does not reuse that operating point and instead follows the values reported in the main manuscript throughout.

Table S7: Predictions for literature-derived external BBB-related peptide candidates that were absent by exact sequence match from both the positive and negative pools used in this study. All predictions were generated with the frozen final pipeline and the same threshold ($\tau = 0.215$) used in the main manuscript.

Candidate	Sequence	Evidence tier	Probability	Prediction	Source	Experimental support summary
RAP12	EAKIEKHNHYQK	High	0.2377	BBB+	PMID 29679668	Reported to facilitate BBB/BBTB penetration of PEG–PLA micelles in vitro and in vivo for glioma-targeted delivery.
PB5-3	QFAALPVRAHYG	High	0.4489	BBB+	PMID 34998171	Reported to enhance AAV9 crossing of the BBB after systemic administration and to increase transcytosis in hCMEC/D3 cells.
M1	TFYGGRPKRNNF LRGIR	Medium	0.9556	BBB+	PMID 31615203	Reported as a BBB/BBTB-penetrating peptide from phage-display screening and used for glioma- and brain-metastasis-targeted therapeutic delivery.
NFL-TBS.40-63	YSSYSAPVSSSL SVRRSYSSSSGS	Medium	0.2486	BBB+	PMID 37722495	Reported to cross an in vitro BBB model and to improve liposome uptake after BBTB passage in a glioblastoma-focused setting.
